# Associations of Social Isolation with Anxiety and Depression During the Early COVID-19 Pandemic: A Survey of Older Adults in London, UK

**DOI:** 10.3389/fpsyt.2020.591120

**Published:** 2020-09-17

**Authors:** Catherine E. Robb, Celeste A. de Jager, Sara Ahmadi-Abhari, Parthenia Giannakopoulou, Chinedu Udeh-Momoh, James McKeand, Geraint Price, Josip Car, Azeem Majeed, Helen Ward, Lefkos Middleton

**Affiliations:** ^1^ Ageing Epidemiology Research Unit, School of Public Health, Imperial College London, London, United Kingdom; ^2^ Department of Primary Care and Public Health, Imperial College London, London, United Kingdom; ^3^ Centre for Population Health Sciences, Lee Kong Chian School of Medicine, Nanyang Technological University, Singapore, Singapore; ^4^ Imperial College Healthcare NHS Trust, Faculty of Medicine, Imperial College London, Public Health Directorate, London, United Kingdom; ^5^ Department of Infectious Disease Epidemiology, School of Public Health, Imperial College London, London, United Kingdom

**Keywords:** ****COVID-19, older adults, anxiety, depression, mental health, social isolation, loneliness

## Abstract

The COVID-19 pandemic is imposing a profound negative impact on the health and wellbeing of societies and individuals, worldwide. One concern is the effect of social isolation as a result of social distancing on the mental health of vulnerable populations, including older people. Within six weeks of lockdown, we initiated the CHARIOT COVID-19 Rapid Response Study, a bespoke survey of cognitively healthy older people living in London, to investigate the impact of COVID-19 and associated social isolation on mental and physical wellbeing. The sample was drawn from CHARIOT, a register of people over 50 who have consented to be contacted for aging related research. A total of 7,127 men and women (mean age=70.7 [SD=7.4]) participated in the baseline survey, May–July 2020. Participants were asked about changes to the 14 components of the Hospital Anxiety Depression scale (HADS) after lockdown was introduced in the UK, on 23^rd^ March. A total of 12.8% of participants reported feeling worse on the depression components of HADS (7.8% men and 17.3% women) and 12.3% reported feeling worse on the anxiety components (7.8% men and 16.5% women). Fewer participants reported feeling improved (1.5% for depression and 4.9% for anxiety). Women, younger participants, those single/widowed/divorced, reporting poor sleep, feelings of loneliness and who reported living alone were more likely to indicate feeling worse on both the depression and/or anxiety components of the HADS. There was a significant negative association between subjective loneliness and worsened components of both depression (OR 17.24, 95% CI 13.20, 22.50) and anxiety (OR 10.85, 95% CI 8.39, 14.03). Results may inform targeted interventions and help guide policy recommendations in reducing the effects of social isolation related to the pandemic, and beyond, on the mental health of older people.

## Introduction

With unprecedented population aging; the consequences of social isolation on the mental wellbeing of older people is emerging as a significant public health concern, now exacerbated by the COVID-19 pandemic (1, 2). Previous studies have reported that social disconnection puts older people at greater risk of depression and anxiety (3). The impact of severe acute respiratory syndrome (SARS) on mental health, within the general public has previously been reported (4), and recent systematic reviews are beginning to highlight the detrimental impact of COVID-19 on mental health among different populations (5–7). Factors exacerbating this risk are less known but vital in informing appropriate targeted intervention and preventative measures.

The United Kingdom (UK) announced COVID-19 lockdown measures on the 23^rd^ March 2020. Lockdown stipulated a ban on nonessential travel, closure of most shops, offices and public spaces, alongside self-isolation and quarantine for those with possible infection and shielding for those deemed extremely vulnerable due to health conditions. These measures have placed many individuals under conditions of complete isolation, especially those living alone. Long periods of social isolation may have a profound negative effect on mental health conditions including depression, anxiety, stress and insomnia (8), may differ as a function of sex and age (5), and may worsen health inequalities, with poorer and marginalized groups at greatest risk (9). Furthermore, social isolation, loneliness and depression have, in turn, been associated with cognitive decline (10, 11) and incident dementia (12, 13) among older people.

A systematic review, conducted in May 2020, sought to identify the psychiatric symptoms or morbidities associated with COVID-19 among those infected, the general population, psychiatric patients and health-care workers (5). They identified 43 studies, the majority of which were conducted within Chinese populations, investigating the impact of COVID-19 on mental health, but not exclusive to the elderly. One Danish study (n=2,458), conducted within the general public, revealed higher scores in anxiety and depression when compared to pre-lockdown (14), especially among females, while a Chinese study (n=333) reported a moderate-to-severe level of subjective stress, anxiety and depression in an initial survey post-lockdown, with no significant changes one-month later (15). Another systematic review and meta-analysis was conducted on studies relating to the mental health impact of COVID-19 on the general public and health workers, up until the 25^th^ May, including 65 studies, again, predominantly from China (7). They reported the prevalence of anxiety and depression among the general population during the pandemic as 33% (28%–30%) and 28% (23%–32%), respectively. Common risk factors for higher psychological impact included being female, having contracted COVID-19, lower socio-economic status, social isolation and spending longer watching COVID-19 related news. Frontline providers of telephone help services such as Lifeline in Australia, have reported dramatic increases in calls from people experiencing anxiety and loneliness (16). The Australian Bureau of Statistics’ national Household impacts of COVID-19 survey of 1000 adults found that 28% of women and 16% of men reported feeling lonely as a result of the pandemic, and that this was the most common personal stressor identified (17). Finally, a UK study has published findings on the impact of COVID-19 on mental health before and during the pandemic, in participants of the UK Household Longitudinal Study (aged >16 years, n=17,452) (18). A web-based survey administered between April 23–30^th^ 2020, assessed mental health *via* the 12-item General Health Questionnaire and reported that prevalence of mental health distress rose from 18.9% (17.8, 20.0) in 2018–2019 to 27.3% (26.3, 28.2) in April 2020. Predictors of change were greatest in younger adults, women and people living with children.

Among these studies, the older population is largely underrepresented. We are not aware of any studies in high income countries that have exclusively investigated the impact of social isolation and physical distancing due to COVID-19 restrictions on the mental health of older people. Identifying the key factors that place older people at risk of decline in mental wellbeing is critical in planning appropriate mitigation strategies. Here, we report the effects of social isolation on self-reported changes in levels of depression and anxiety among older people residing within London *via* an online survey. We investigated the effect of sociodemographic factors, health variables and indicators of loneliness and reduced connectivity as risk factors for change in levels of depression and anxiety. As the literature presents consistent evidence for the effect modification of sex in response to social isolation on mental health (14, 19–23), we also explored whether certain risk factors differentially altered responses to social isolation among men and women. Results may inform interventions to prevent or delay the effects of social isolation on worsening mental health in this susceptible older population.

## Materials and Methods

### Study Design and Population

To investigate the associations between social isolation measures, implemented due to the COVID-19 pandemic, and the mental and physical health of an older population, we designed and implemented, on April 29^th^, 2020, the ongoing longitudinal CHARIOT COVID-19 Rapid Response Study (CCRR). Study participants were recruited from the Cognitive Health in Ageing Register for Interventional and Observation Trials (CHARIOT), comprised of ~40,000 volunteers aged 50 years and over, without known dementia diagnosis and who have consented to be contacted for participation in age-related research (24). CHARIOT has been developed by the School of Public Health at Imperial College London, since 2012, in collaboration with primary care practices and community organizations across London. For the CCRR study, data on symptoms and results of COVID-19 tests, demographic and lifestyle factors, mental and physical health are being collected by repeated six-weekly questionnaire online surveys. In the present analysis we report cross-sectional results from the baseline survey, conducted between 30^th^ April – 8^th^ July 2020. All register volunteers were invited *via* email or post for participation in the CCRR study. Additional adult members of their household, able to provide consent and who wished to take part in the survey, could do so by contacting the study team. Participants were directed *via* a unique link to the online survey platform, hosted by Qualtrics (Provo, UT, USA), where they were presented with the Participant Information Sheet, then directed to complete an electronic Informed Consent Form. Once the consent form was electronically signed by the participant, the survey was launched. Data collected as a part of this study are anonymized and kept strictly confidential in accordance with the UK General Data Protection Regulations (2016). CCRR was ethically approved by the Imperial College London Joint Research Compliance Office (20IC5942) and by the Health Research Authority (16/EM/0213).

### Assessment of Sociodemographic, Health and Lifestyle Factors

Data on general (age, sex, ethnicity, and marital status) demographics, household composition, current occupational status and friend/family contact *via* technology such as skype/zoom/mobile were extracted. Alcohol and smoking behavior, and height/weight for the calculation of body mass index (BMI) were included. Participants were asked to report any medical history *via* checking against a list of comorbidities including vascular factors, cancers, neurological and mental health conditions, arthritis and respiratory disease. Loneliness was measured *via* the following question: “During the period of reduced social contact, have you experienced loneliness (felt isolated, with no companions)”, with the following responses; “never”, “rarely”, “sometimes”, “often”. The variable used to assess sleep was obtained from the question: “During the period of reduced social contact, have you experienced poor sleep (restless and unable to sleep)”, with the following responses; “Not ever”, “Less than once a week”, “Once or twice a week”, “Three or more times a week”. The sleep and loneliness questions were obtained from the Imperial College Sleep Quality questionnaire adapted from the Pittsburgh Sleep Quality Index (25) and Centre for Epidemiologic Studies of Depression Scale, for work-free periods (26).

### Depression and Anxiety (Outcome)

**Figure 1:**
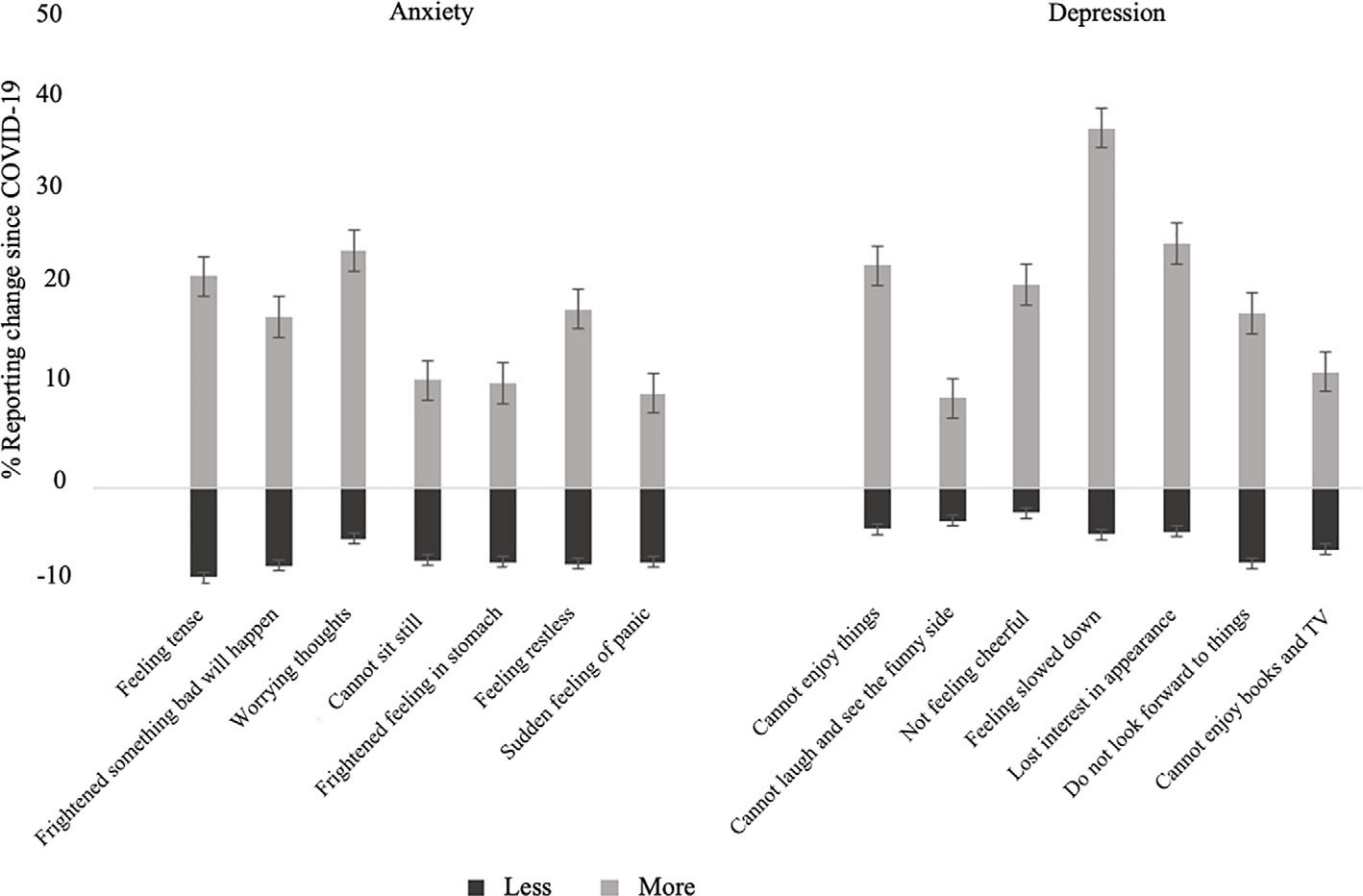
Percent of cohort reporting worsened or improved items of anxiety and depression following COVID-19 social isolation measures. Error bars indicate standard error.

Depression and anxiety levels were assessed with the Hospital Anxiety and Depression Scale (HADS) which includes 14 questions on feelings related to anxiety and depression (seven items for each), rated on a 4-level Likert scale from “most of the time” to “not at all” or similar responses (27). The widely used HADS has face validity for use in an older population (28), with questions that are easy to relate to and appropriate to the current circumstances of social isolation. After each item, we added a question as to whether participants were experiencing that feeling “more than”, “less than” or “the same as” before COVID-19 social distancing restrictions. The categorical outcome variable used in this study was overall improvement, worsening or no change in reported items of anxiety and depression (**Figure 1**). Participants were categorized as either worsened or improved on the depression or anxiety components of HADS if they responded feeling “more than” or “less than” since before lockdown, on four or more of the seven items for depression or anxiety, respectively. All others were categorized as not changed.

### Statistical Analyses

We conducted separate multinomial logistic regression models to assess the association between each of the following factors: sex (men, women), age (continuous, years), marital status (married/partnered, single/widowed/divorced), smoking (no, yes), alcohol consumption (continuous, units per week), sleep quality (not ever, < once per week, 1–2 times per week, ≥3-times per week), feelings of loneliness (never, rarely, sometimes, often), household composition (not living alone, living alone), level of remote friend/family contact *via* technology (daily, 2–6 times per week, ≤ once per week) and their association with risk of change in components of anxiety and depression since lockdown as separate outcomes (worsened, improved, no change). Analyses was initially conducted in men and women combined, followed by sex-stratification. Interaction terms by sex and exposure were included in each model to determine if the effect of exposure on the outcome measure significantly varied as a function of sex. Models were controlled for confounding effects of age and sex (model 1), and additionally for hypertension, hypercholesterolemia, diabetes, cardiovascular disease, chronic obstructive pulmonary disease (COPD) and any mental health conditions, pre-lockdown (model 2). These common chronic conditions were included as subjectively reported poor health is a known risk factor for depression and anxiety (29–31). Less than 7% of data were missing for any one variable; hence, we did not compute missing values. All variables were included in the model as categorical, with the exceptions of age and alcohol consumption. To enhance interpretation of the logistic regression, alcohol consumption was adjusted to represent risk per increase in 3-units of alcohol per week (approximately one glass of wine), and for age, an increase in risk per 5-years. Results are presented as odds ratios (ORs) and 95% confidence intervals (CI). Statistical two-sided significance level was set at 5% (*p*<0.05). All analyses were conducted using IBM SPSS 23 for windows.

## Results

### Cohort Characteristics

At time of data extraction, a total of 9,314 register participants had read the Participant Information Sheet and were directed to complete the consent form. Of this number, 2,187 (24.5%) participants did not complete consent to join CCRR. The remaining 7,127 were included in this study for baseline data analysis. The response rates from 15,000 emailed invitations and 25,000 postal invitations were approximately 35% and 7.5%, respectively.

**Table 1** presents the cohort characteristics. Of the total sample, majority were Caucasian (91.5%) with a mean age 70.6 (SD 7.4) years (range 50-100). Women represented 54.1% of the cohort, 65.5% were married/partnered, and 20.7% were employed. Mean BMI was 25.1 (SD 5.7), 77.1% of men and 50.6% women reported at least one vascular factor, 2.4% of the overall cohort reported a mental health condition, pre-pandemic. Poor sleep ≥3 times per week was reported by 12.3% of men and 20.9% of women. Majority of the cohort reported that they did not smoke (93.6%), and alcohol consumption was low. A higher proportion of men reported feeling lonely “often” and having contact with friends and family ≥3 times per week compared to women; whereas a higher proportion of women reported living alone. A total of 5.5% of participants fell within the abnormal category for anxiety and 2.5% for depression on the HADS questionnaire, according to population norms. Since lockdown, 12.8% of participants reported feeling worse on components of depression on the HADS and 12.3% reported feeling worse on components of anxiety. On the other hand, fewer participants reported feeling improved on components of depression and anxiety (**Figure 1**). There was a substantially higher proportion of women scoring abnormal on the HADS depression and anxiety assessment, and who reported feeling worse in components of anxiety and depression post-lockdown, compared to men.

**Table 1:** Descriptive statistics within the overall cohort and stratified by sex.

	Overall Cohort	Males	Females
**Demographics**			
Sex, n (% women)	7,127 (100)	3,114 (43.7)	3,855 (54.1)
Age, mean years, (SD)	70.6 (7.4)	71.3 (7.2)	70.1 (7.5)
Marital status, n, % married/partner	4,668 (65.5)	2457 (78.9)	2210 (57.3)
Ethnicity, n (%)			
Caucasian	6,522 (91.5)	2,900 (93.1)	3,614 (93.7)
African/Caribbean	48 (0.7)	17 (0.5)	31 (0.8)
Asian	195 (2.7)	98 (3.1)	95 (2.5)
Other	192 (2.7)	89 (2.9)	103 (2.7)
Employment status, n (%)			
Employed	1,444 (20.7)	690 (22.2)	754 (19.5)
Retired	4,815 (67.6)	2,179 (70)	2,633 (68.3)
Furloughed/unemployed	403 (5.6)	155 (5)	241 (6.4)
**Health and Lifestyle**			
BMI, mean (SD)	25.1 (5.7)	26.7 (6.1)	23.9 (5.1)
Normal (<25 kg/m^2^), n (%)	1,272 (57.2)	415 (43.9)	855 (67)
Overweight (25-29.9 kg/m^2^), n (%)	599 (26.9)	299 (31.6)	300 (23.5)
Obese (≥ 30 kg/m^2^), n (%)	352 (15.8)	231 (24.4)	121 (9.5)
Medical history, n (%)			
Hypertension	1,919 (26.9)	1,009 (32.4)	897 (23.3)
Hypercholesterolaemia	1,529 (21.5)	774 (24.9)	743 (19.3)
Arthritis	1,048 (14.7)	352 (11.3)	691 (17.9)
Cardiovascular disease	478 (6.7)	319 (10.2)	154 (4.0)
Type 2 diabetes	456 (6.4)	299 (9.6)	153 (4.0)
Asthma	445 (6.2)	193 (6.2)	250 (6.5)
COPD	198 (2.8)	108 (3.5)	88 (2.3)
Psychiatric diagnosis	173 (2.4)	61 (2.0)	109 (2.8)
Active cancer treatment	114 (1.6)	71 (2.3)	43 (1.1)
Poor sleep, n (%)			
Not ever	1,411 (21.3)	839 (28.3)	571 (15.7)
< once per week	2,501 (37.1)	1,152 (38.9)	1,344 (36.9)
1-2 times per week	1,574 (23.8)	606 (20.5)	966 (26.5)
≥3 times per week	1,125 (17)	365 (12.3)	759 (20.9)
Smoking status, n, % nonsmoker	6,668 (93.6)	2,970 (95.4)	3,693 (95.8)
Alcohol unit’s p/w, median (IQR)	8.0 (2.0, 15.0)	10.0 (3.0, 20.0)	6.0 (1.0, 14.0)
**Indicators of isolation**			
Feeling lonely, n (%)	6,617 (92.8)	2,965 (95.2)	3,643 (94.5)
Not ever	3,245 (49)	1,793 (60.5)	1,449 (39.8)
Rarely	1,573 (23.8)	647 (21.8)	925 (25.4)
Sometimes	1,374 (20.8)	423 (14.3)	947 (26.0)
Often	425 (6.4)	102 (3.4)	322 (8.8)
Household members, n (% living alone)	1,915 (26.9)	571 (18.3)	1,334 (34.6)
Friend/family social media contact, n (%)			
Daily	3,709 (53.7)	1,496 (48.7)	2,249 (59.3)
2-6 times per week	2,415 (35.0)	1,181 (38.5)	1,220 (32.2)
≤ once per week	780 (11.3)	454 (14.8)	320 (8.4)
**Mood**			
HADS Depression score, n (%)			
Normal	5,114 (90.8)	2,368 (93.7)	2,740 (88.5)
Borderline	375 (6.7)	116 (4.6)	258 (8.3)
Abnormal	142 (2.5)	44 (1.7)	98 (3.2)
HADS Anxiety score, n (%)			
Normal	4,774 (84.8)	2,276 (90.0)	2,495 (80.6)
Borderline	550 (9.8)	162 (6.4)	384 (12.4)
Abnormal	307 (5.5)	90 (3.6)	217 (7.0)
Depression change, n (%)^a^			
Same	5,640 (79.1)	2,689 (84.4)	2,946 (76.4)
Worsened	912 (12.8)	243 (7.8)	668 (17.3)
Improved	108 (1.5)	48 (1.5)	60 (1.6)
Anxiety change, n (%)^a^			
Same	5,432 (76.2)	2,612 (83.9)	2,815 (73.0)
Worsened	880 (12.3)	244 (7.8)	636 (16.5)
Improved	348 (4.9)	124 (4.0)	223 (5.8)

a Denotes change in ≥4 items within the seven mood items (each for depression and anxiety).

SD, standard deviations; kg, kilograms; m^2^, meters squared; BMI, body mass index; COVID, 2019 coronavirus pandemic; COPD, chronic obstructive pulmonary disease; HADS, Hospital Anxiety and Depression Scale.

### Association of Age and Sex With Change in Anxiety and Depression

After accounting for the confounding effect of covariates, women compared to men were more than twice as likely to report feeling worse on components of depression (OR 2.46, 95% CI 2.10, 2.89) and anxiety (OR 2.42, 95% CI 2.06, 2.85) on the HADS (**Tables 2**, **3**). Conversely, of those reporting improvements (4.9%), women were more likely to report feeling better in components of anxiety (OR 1.7, 95% CI 1.36, 2.16), relative to men. With every five-year increase in age there was a 19% (OR 0.81, 95% CI 0.77, 0.85) and 22% (OR 0.78, 95% CI 0.75, 0.83) lower risk of reporting feeling worse on components of depression and anxiety, respectively.

**Table 2:** Association between sociodemographic factors, health and lifestyle, indicators of isolation and change in components of depression.

Predictor		Worsened, OR [95% CI]		Improved, OR [95% CI]	
	*N*	Model 1	Model 2	Model 1	Model 2
Sex, ref. Men	6581				
Women		2.43 [2.07, 2.84]^***^	2.46 [2.10, 2.89]^***^	1.10 [0.75, 1.62]	1.14 [0.77, 1.69]
Age (years)	6581	0.81 [0.77, 0.85]^***^	0.81 [0.77, 0.85]^***^	0.88 [0.76, 1.00]	0.89 [0.78, 1.02]
Marital status, ref. Married/partner	6580				
Single/widow/divorced		1.40 [1.20, 1.63]^***^	1.37 [1.17, 1.59]^***^	0.68 [0.43, 1.08]	0.65 [0.41, 1.03]
Smoking status, ref. Nonsmoker	6580				
Smoker		1.50 [1.04, 2.16]^*^	1.41 [0.97, 2.04]	2.26 [1.03, 4.94]^*^	2.07 [0.94, 4.57]
Alcohol consumption (units p/w)	6580	1.02 [1.00, 1.04]^*^	1.01 [1.00, 1.01]^*^	0.98 [0.66, 1.44]	0.97 [0.95, 0.99]^**^
Poor sleep, ref. Not ever	6535				
< once per week		2.00 [1.51, 2.64]^***^	2.00 [1.51, 2.65]^***^	0.71 [0.43, 1.16]	0.72 [0.44, 1.18]
1-2 times per week		2.85 [2.14, 3.79]^***^	2.84 [2.13, 3.79]^***^	0.79 [0.46, 1.36]	0.78 [0.45, 1.35]
≥3 times per week		7.11 [5.37, 9.41]^***^	6.91 [5.21, 9.15]^***^	0.80 [0.43, 1.51]	0.75 [0.40, 1.42]
Feeling lonely, ref. Not ever	6535				
Rarely		2.72 [2.16, 3.42]^***^	2.72 [2.16, 3.43]^***^	0.61 [0.37, 1.02]	0.62 [0.37, 1.02]
Sometimes		7.22 [5.84, 8.92]^***^	7.14 [5.78, 8.82]^***^	0.51 [0.27, 0.94]^*^	0.49 [0.26, 0.91]^*^
Often		18.34 [14.09, 23.86]^***^	17.24 [13.20, 22.50]^***^	0.97 [0.39, 2.45]	0.77 [0.30, 1.99]
Household members, ref. Not alone	6580				
Live alone		1.36 [1.16, 1.60]^***^	1.32 [1.12, 1.55]^**^	0.65 [0.39, 1.07]	0.62 [0.37, 1.02]
Friend/family social media contact, ref. Daily	6534				
2-6 times per week		1.04 [0.89, 1.21]	1.05 [0.90, 1.23]	0.71 [0.49, 1.09]	0.72 [0.47, 1.11]
≤ once per week		1.03 [0.81, 1.32]	0.99 [0.77, 1.27]	0.72 [0.37, 1.41]	0.69 [0.35, 1.36]

Reference outcome category: No change. p ≤ 0.001^***^, p ≤ 0.01^**^, p ≤ 0.05^*^. Model 1 adjusted for age and sex; Model 2 adjusted for age, sex, hypertension, hypercholesterolemia, type 2 diabetes, chronic obstructive pulmonary disease, cardiovascular disease and mental health conditions prior to lockdown. N; sample size in each multivariable regression model (N did not differ in models 1 and 2).

**Table 3:** Association between sociodemographic factors, health and lifestyle, indicators of isolation and change in components of anxiety.

		Worsened, OR [95% CI]		Improved, OR [95% CI]	
Predictor	*N*	Model 1	Model 2	Model 1	Model 2
Sex, ref. Men	6581	2.33 [1.98, 2.73]^***^	2.42 [0.75, 0.83]^***^	1.63 [1.30, 2.05]^***^	1.7 [1.36, 2.16]^***^
Age (years)		0.79 [0.76, 0.83]^***^	0.78 [0.75, 0.83]^***^	0.96 [0.89, 1.04]	0.97 [0.89, 1.04]
Marital status, ref. Married/partner	6581				
Single/widow/divorced	6580	1.20 [1.02, 1.40]^*^	1.17 [1.00, 1.37]^*^	1.32 [1.04, 1.67]**	1.30 [1.03, 1.64]^*^
Smoking status, ref. Nonsmoker					
Smoker	6580	1.25 [0.85, 1.85]	1.16 [0.79, 1.72]	1.39 [0.79, 2.42]	1.36 [0.78, 2.38]
Alcohol consumption (units p/w)		1.01 [0.99, 1.03]	1.00 [1.00, 1.01]	0.99 [0.96, 1.02]	1.00 [0.99, 1.01]
Poor sleep, ref. Not ever	6580				
< once per week	6535	1.80 [1.33, 2.44]^***^	1.81 [1.34, 2.45]^***^	0.52 [0.40, 0.68]^***^	0.53 [0.41, 0.69]^***^
1-2 times per week		3.52 [2.61, 4.76]^***^	3.50 [2.59, 4.73]^***^	0.44 [0.32, 0.60]^***^	0.43 [0.31, 0.60]^***^
≥3 times per week		7.90 [5.87, 10.63]^***^	7.67 [5.69, 10.33]^***^	0.42 [0.29, 0.62]^***^	0.41 [0.28, 0.61]^***^
Feeling lonely, ref. Not ever					
Rarely	6535	1.65 [1.32, 2.07]^***^	1.65 [1.32, 2.07]^***^	0.73 [0.55, 0.96]^*^	0.72 [0.56, 0.96]^*^
Sometimes		4.77 [3.91, 5.82]^***^	4.73 [3.87, 5.77]^***^	0.63 [0.46, 0.88]^**^	0.62 [0.45, 0.86]^**^
Often		11.27 [8.75,14.51]^***^	10.85 [8.39, 14.03]^***^	0.58 [0.31, 1.10]	0.53 [0.28, 0.99]^*^
Household members, ref. Not alone					
Live alone	6580	1.89 [1.01, 1.40]^*^	1.15 [0.98, 1.36]	1.07 [0.83, 1.37]	1.05 [0.82, 1.35]
Friend/family social media contact, ref. Daily					
2-6 times per week	6534	0.79 [0.68, 0.93]^**^	0.81 [0.68, 0.95]^**^	0.73 [0.57, 0.93]^**^	0.74 [0.57, 0.94]^*^
≤ once per week		0.79 [0.61, 1.02]	0.77 [0.59, 1.00]	0.77 [0.52, 1.13]	0.76 [0.51, 1.11]

Reference outcome category: No change. p ≤ 0.001^***^, p ≤ 0.01^**^, p ≤ 0.05^*^. Model 1 adjusted for age and sex; Model 2 adjusted for age, sex, hypertension, hypercholesterolemia, type 2 diabetes, chronic obstructive pulmonary disease, cardiovascular disease and mental health conditions prior to lockdown. N; sample size in each multivariable regression model (N did not differ in models 1 and 2).

### Loneliness and Reduced Social Connectivity

Overall, 27.2% of the cohort reported that they felt lonely sometimes or often, more in women (34.8%) than men (17.7%). There was a prominent and dose-response association between loneliness and worsened components of anxiety and depression on the HADS. Individuals reporting that they “often” felt lonely had a 17.24 (95% CI 13.20, 22.50) times higher risk of reporting feeling worse in components of depression and 10.85 (95% CI 8.39, 14.03) times higher risk of reporting feeling worse in components of anxiety, compared to those who never felt lonely (**Tables 2**, **3**). Women were twice as likely to report worsened components of depression as a result of loneliness (OR 19.74, 95% CI 14.28, 27.29) compared to men (OR 11.60, 95% CI 6.86, 19.62), and men were more likely to report worsened anxiety (OR 14.79, 95% CI 8.99, 24.32) than women (OR 9.36, 95% CI 6.92, 1.80) (**Tables 4**, **5**).

**Table 4:** Association between sociodemographic factors, health and lifestyle, indicators of isolation and change in components of depression among males and females.

	Worsened, OR [95% CI]		Improved, OR [95% CI]	
Predictor	Male	Female	Male	Female
Age (years)	0.83 [0.75, 0.91]^***^	0.80 [0.76, 0.85]^***^	1.10 [0.89, 1.35]	0.78 [0.65, 0.93]^**^
Marital status, ref. Married/partner				
Single/widow/divorced	1.73 [1.28, 3.34]^***^	1.26 [1.06, 1.51]^**^	0.78 [0.36, 1.69]	0.64 [0.36, 1.15]
Smoking status, ref. Nonsmoker				
Smoker	1.75 [0.97, 3.14]	1.24 [0.77, 1.99]	1.17 [0.28, 4.99]	2.95 [1.12, 7.79]^*^
Alcohol consumption (units p/w)	1.01 [1.00, 1.02]	1.01 [1.00, 1.01]	0.98 [0.96, 1.01]	0.78 [0.66, 0.93]^**^
Poor sleep, ref. Not ever				
< once per week	2.32 [1.47, 3.69]***	1.82 [1.27, 2.59]***	1.38 [0.66, 3.76]	0.39 [0.20, 0.75]^**^
1-2 times per week	3.15 [1.93, 5.13***	2.64 [1.84, 3.77]***	1.61 [0.70, 3.70]	0.44 [0.21, 0.90]^*^
≥3 times per week	7.68 [4.76, 12.39]***	6.33 [4.45, 9.00]***	1.13 [0.38, 3.33]	0.52 [0.24, 1.13]
Feeling lonely, ref. Not ever				
Rarely	2.86 [1.97, 4.16]^***^	2.68 [2.00, 3.60]^***^	0.88 [0.43, 1.80]	0.48 [0.24, 0.97]^*^
Sometimes	7.53 [5.28, 10.74]^***^	7.17 [5.49, 9.37]^***^	0.30 [0.07, 1.27]	0.55 [0.27, 1.12]
Often	11.60 [6.86, 19.62]^***^	19.74 [14.28, 27.29]^***^	1.59 [0.44, 5.71]	0.46 [0.11, 1.98]
Household members, ref. Not alone				
Live alone	1.61 [1.17, 2.21]^**^	1.25 [1.03, 1.50]^*^	0.87 [0.40, 1.88]	0.55 [0.28, 1.06]
Friend/family social media contact, ref. Daily				
2-6 times per week	0.94 [0.70, 1.25]	1.09 [0.90, 1.32]	1.10 [0.60, 2.02]	0.46 [0.24, 0.90]^*^
≤ once per week	0.78 [0.51, 1.19]	1.13 [0.83, 1.55]	0.69 [0.26, 1.84]	0.82 [0.32, 2.11]

Reference outcome category: No change. p ≤ 0.001^***^, p ≤ 0.01^**^, p ≤ 0.05^*^. Adjusted for age, hypertension, hypercholesterolemia, type 2 diabetes, chronic obstructive pulmonary disease, cardiovascular disease and mental health conditions prior to lockdown.

**Table 5:** Association between sociodemographic factors, health and lifestyle, indicators of isolation and change in components of anxiety among males and females.

Predictor	Worsened, OR [95% CI]		Improved, OR [95% CI]	
	Male	Female	Male	Female
Age (years)	0.79 [0.72, 0.87]^***^	0.78 [0.74, 0.83]^***^	0.97 [0.85, 1.10]	0.97 [0.88, 1.06]
Marital status, ref. Married/partner				
Single/widow/divorced	1.44 [1.06, 1.97]^*^	1.09 [0.91, 1.32]	1.20 [0.78, 1.84]	1.34 [1.01, 1.78]^*^
Smoking status, ref. Nonsmoker				
Smoker	1.69 [0.95, 3.02]	0.91 [0.54, 1.55]	0.88 [0.31, 2.47]	1.71 [0.87, 3.36]
Alcohol consumption (units p/w)	1.00 [0.99, 1.01]	1.01 [0.20, 1.02]	1.00 [0.99, 1.01]	0.99 [0.98, 1.01]
Poor sleep, ref. Not ever				
< once per week	1.39 [0.87, 2.21]	2.10 [1.39, 3.15]^***^	0.61 [0.40, 0.92]^*^	0.47 [0.33, 0.66]^***^
1-2 times per week	3.45 [2.19, 5.44]^***^	3.66 [2.44, 5.49]^***^	0.40 [0.22, 0.73]^*^	0.42 [0.29, 0.63]^***^
≥3 times per week	7.58 [4.80, 11.98]^***^	8.07[5.40, 12.05]^***^	0.63 [0.33, 1.19]	0.33 [0.20, 0.54]^***^
Feeling lonely, ref. Not ever				
Rarely	2.11 [1.45, 3.08]^***^	1.43 [1.08, 1.90]^**^	0.77 [0.48, 1.23]	0.70 [0.50, 0.99]^*^
Sometimes	5.71 [4.03, 8.08]^***^	4.21 [3.30, 5.37]^***^	0.50 [0.25, 1.00]^*^	0.66 [0.45, 0.95]^*^
Often	14.79 [8.99, 24.32]^***^	9.36 [6.92, 1.80]^***^	0.96 [0.32, 2.82]	0.42 [0.19, 0.91]^*^
Household members, ref. Not alone				
Live alone	1.52 [1.11, 2.09]^**^	1.05 [0.86, 1.27]	0.96 [0.59, 1.54]	1.08 [0.81, 1.46]
Friend/family social media contact, ref. Daily				
2-6 times per week	0.66 [0.49, 0.88]^**^	0.88 [0.72, 1.07]	0.49 [0.32, 0.75]^***^	0.92 [0.68, 1.25]
≤ once per week	0.55 [0.37, 0.87]^*^	0.92 [0.67, 1.28]	0.55 [0.31, 0.99]^*^	0.97 [0.58, 1.62]

Reference outcome category: No change. p ≤ 0.001^***^, p ≤ 0.01^**^, p ≤ 0.05^*^. Adjusted for age, hypertension, hypercholesterolemia, type 2 diabetes, chronic obstructive pulmonary disease, cardiovascular disease and mental health conditions prior to lockdown.

Compared to those who reported living with others, those who lived alone were more likely to report feeling worse on components of anxiety (OR 1.89, 95% CI 1.01, 1.40) and depression (OR 1.36, 95% CI 1.16, 1.60) (**Tables 2**, **3**). Findings were augmented among men (**Tables 4**, **5**). The associations were attenuated but remained significant after accounting for the confounding effect of self-reported mental health conditions and vascular factors.

Level of remote contact with friends/family *via* technology did not significantly alter risk of reporting feeling worse on components of depression (**Tables 2**, **4**). Compared to individuals who reported daily contact, those reporting 2–6 times of online social contact per week had a 19% (OR 0.81, 95% CI 0.68, 0.95) lower risk of reporting feeling worse on components of anxiety, and, conversely, a 26% (OR 0.74, 95% CI 0.57, 0.94) lower likelihood of reporting feeling improved (**Table 3**). Sex stratified analysis found these results to be augmented and to remain statistically significant among men (**Table 5**).

Single/widowed/divorced individuals had a 1.37 (95% CI 1.17, 1.59) and 1.17 (95% CI 1.00, 1.37) times higher risk of reporting worsened components of depression and anxiety on the HADS, respectively, compared to those who were married/partnered (**Tables 2**, **3**). These associations were augmented among men (**Tables 4**, **5**). There was also a small proportion more likely to report feeling improvement on components of anxiety, following lockdown (OR 1.30, 95% CI 1.03, 1.64), compared to those who are married/partnered, which were augmented among women (**Table 5**).

### Sleep, Alcohol, and Smoking

Male smokers were more likely to report feeling worse on components of depression (OR 1.75, 95% CI 0.97, 3.14) and anxiety (OR 1.69, 95% CI 0.95, 3.02) on the HADS compared to nonsmokers (**Tables 4**, **5**). This association was not significant for women. However, of those reporting improvements in components of depression, female smokers were more likely to do so than female nonsmokers, while this association was not statistically significant for men. Alcohol consumption was not associated with a remarkable worsening or improvement in components of anxiety or depression in men. However, a three-unit increase in alcohol consumption per week (approximately one glass of wine) was associated with a 22% (OR 0.78, 95% CI 0.66, 0.93) lower likelihood of reporting improvement in components of depression in women.

Cohort participants who subjectively reported experiencing poor sleep were more likely to report worsened components of anxiety and depression and less likely to report improvement, in a dose response manner. Those reporting poor sleep ≥3 times per week had a 6.91 (95% CI 5.21, 9.15) and 7.67 (95% CI 5.69, 10.33) times higher risk for reporting feeling worse in components of depression and anxiety, respectively, compared to those who reported an absence of poor sleep (**Tables 2**, **3**). Differences did not vary significantly by sex.

## Discussion

We investigated the effect of sociodemographic, health and lifestyle factors, indicators of loneliness and reduced connectivity on subjective feelings of anxiety and depression among an older population. Most people did not report a change on components of anxiety and depression on the HADS, but for those who did report change, it was more likely worsened than improved (**Table 1**). Our results indicate that women, younger age, being single/widowed/divorced, living alone, poor sleep and experiencing loneliness are factors linked with higher risk for reporting worsened components of anxiety and/or depression.

### Loneliness and Reduced Social Connectivity

This study demonstrated a significant negative association between subjective loneliness and worsened components of both depression and anxiety, following lockdown. These associations had a dose response effect. Levels of anxiety were exacerbated among men, and depression, among women. Furthermore, descriptive statistics indicated a significant change in loneliness before and after lockdown stipulations, whereby those reporting loneliness “often” prior to lockdown increased from 2% to 20% post-lockdown (data not shown). These findings indicate that an increase in loneliness was most likely due to the circumstances surrounding COVID-19 social isolation and was not pre-existing. Our findings corroborate results from a survey on the impact of COVID-19 on mental health (32), as reported in a recent Lancet Psychiatry position paper, indicating a strong association between social isolation and loneliness with symptoms of depression and anxiety (33). Social isolation and loneliness are strongly associated with anxiety, depression, self-harm and suicide attempts across the lifespan (34–36). Older people may be considered prime candidates for risk of loneliness, owing to the higher likelihood of reduced capacity, frailty and comorbidities, and reduced likelihood to engage with others *via* technology. Our results found that those who were single/widowed/divorced and/or who lived alone were also at increased risk of reporting worsened components of depression and anxiety following COVID-19 lockdown, especially among men. Furthermore, men who engaged in higher levels of friend/family contact *via* technology, reported feeling worse in components of anxiety, perhaps indicating reverse causality. Being widowed or divorced as a risk factor for worsened mental health has been reported in similar COVID-19 general population cohort studies in Spain (n=3,055) (19) and China (n=1,060) (37), although among younger cohorts and without investigating the effect modification of sex. It may be expected that living alone and without a partner are inherently linked with an increased risk of loneliness, especially under circumstances of social and physical distancing. The frequency and mode of social connectivity *via* technology, while under social distancing circumstances, and its link with anxiety and depression has not yet been investigated outside the current study, warranting further attention.

The longer-term consequence of such risk factors as loneliness and reduced social connectivity have been reported elsewhere. Social isolation, depression and apathy have been associated with an increased risk of incident dementia in a circular-causal manner (12, 38). Furthermore, data from the English Longitudinal Study of Ageing reported that incident dementia was independently associated with loneliness, a lower number of close relationships and not being married, and that these findings were in fact independent of depression and without reverse causality (13). Our findings and those reported above, only further highlight the need to promptly tackle both the immediate and longer-term consequence of social isolation on the mental and consequential cognitive health of older adults.

### Sleep, Alcohol, and Smoking

A total of 40% of our cohort reported sleep disturbances. This figure exceeds worldwide insomnia prevalence, estimated before the pandemic to be between 3.9% and 22% (39). A study conducted in Greece (n=2,427), following COVID-19 lockdown, detected a similar proportion (37.6%) of the general public experiencing some level of sleep disturbance (40). They also reported that women, living in urban areas, stress surrounding risk of COVID-19 infection, loneliness and severe depressive symptoms were all predictive of insomnia. Therefore, it is not unreasonable to suggest that, in such circumstances, sleep disturbances may be an artifact of reverse or bi-directional causality. It may be expected that personal circumstances surrounding the COVID-19 pandemic will increase levels of stress. Worry and ruminating thoughts provoke cognitive arousal and may disturb cortisol homeostasis, resulting in poorer sleep. Such associations have previously been reported under similar circumstances (41). Furthermore, there is existing evidence that loneliness and poor sleep have a bi-directional relationship (42).

We found that men who reported smoking had an increased risk of reporting worsened components of anxiety and depression. Conversely, among the sub-group of those reporting improved components of depression, females with higher alcohol consumption were 22% less likely to report these improvements. Although no study has yet investigated the associations of smoking and alcohol with risk for depression and anxiety during the COVID-19 pandemic, social isolation has been reportedly associated with unhealthy lifestyle factors, including increased smoking and alcohol consumption (35). In our study, of those who smoke, 24.6% reported that they had increased smoking since lockdown, and of drinkers, 14.7% reported an increase in alcohol consumption, both warranting further investigation. Once again, these observations may be a consequence of reverse or bi-directional causality. Nonetheless, majority of participants report no change in smoking and/or drinking behavior post-lockdown, indicating that perceived worsening in components of depression and/or anxiety may also be linked with this pre-existing behavior. Exploration of longitudinal data will elucidate such inferences.

### Age and Sex

Women, compared to men, were more likely to report worsened components of anxiety and depression on the HADS. These findings have been replicated, in varying age-groups and from different countries including the UK (18), Demark (14), Spain (19, 23, 43), Italy (21), Turkey (44) and Iran (20, 22). Furthermore, studies conducted on the effects of stress, have consistently reported women to be at increased risk of developing anxiety and depression (45). Notwithstanding, one recent study reported that associations between depression, stress and insomnia was higher among men surveyed during the COVID-19 pandemic (46), while another study reported no differences related to sex (37), both conducted within Chinese populations. To the best of our knowledge, ours is the first to report the effect modification of sex on the association of key risk factors for depression and anxiety, among older people, during the COVID-19 lockdown. Such findings may elucidate causative variations in risk for mental health decline. Factors including loneliness, being single/widowed/divorced, living alone, remote friend/family contact *via* technology, and alcohol consumption were all contributors to differences between men and women in reported worsening in components of anxiety and depression. These results highlight the importance of investigating specific sociodemographic, health and lifestyle circumstances which augment risk among men and/or women differentially.

In our population of older people, we found that younger age was a risk factor for worsened components of anxiety and depression. To our awareness, only one other study of a much smaller sample (n=236) reported on the associations of COVID-19-related social isolation on mental health among older people exclusively (44), but the authors did not investigate the risk of age. The older age-group is poorly represented within most of reported studies, to date. However, two studies reported lowest risk for anxiety and depression during the earlier stages of COVID-19 lockdown, among a small sub-sample of those >60 years, when compared to younger age-categories, both being within Spanish cohorts (19, 43). Conversely, a Chinese population study, reported that older age increased risk for anxiety and depression (37). Although the effect of COVID-19 on mental health appears to be attenuated by older age, findings within an older sample are scarce and studies have often failed to account for risk factors more commonly affecting older people, such as social isolation and loneliness. Indeed, social disconnection has reportedly put older people at great risk of depression and anxiety (3). Nonetheless, among a healthier older population such as ours, it may also be that with increasing age, older adults are more able to adapt and show higher resilience. To truly understand the relevance of our findings, follow-up data will need to be investigated, and ideally, in comparison with a congruent, younger population.

### Limitations

Some study limitations warrant acknowledgment. Firstly, we did not have a measure of anxiety and depression before the COVID-19 social isolation and physical distancing measures were mandated. Thus, we were unable to assess change other than from current and self-reported change. Nonetheless, given the magnitude of observed outcomes, it is not unreasonable to speculate that mental health changes were largely influenced by circumstances surrounding the COVID-19 pandemic and resultant social isolation. Indeed, by comparing the proportion of those reporting worsened components of anxiety and depression against HADS clinical classification (normal, borderline, abnormal), 53% of those who reported worsened depression, and 34% of those who reported worsened anxiety, scored within the normal range on the HADS scale, indicating that lockdown affected mood not only among those with pre-existing disorders, but also in psychologically healthy individuals.

The use of cross-sectional data in this study precludes causal inferences. We are unable to establish the direction of the association between various factors such as changes in alcohol consumption, cigarette smoking, sleep quality, and worsened levels of anxiety or depression. It will be important to investigate repeated measures of modifiable exposures and reported symptoms of depression and anxiety over time. Nonetheless, the CCRR study is ongoing and we endeavor to publish longitudinal findings in due course. Furthermore, we have not yet captured the experiences of those less technologically literate. Wider access to technology may help buffer loneliness and isolation that lead to worsened mental health. Older people, however, are more likely to have limited ability to access technology, most likely representing the more vulnerable of this demographic. We may hypothesize that those who are less able or willing to engage with technology may also present with exacerbated risk factors such as a higher prevalence of comorbidities, and hence, be yet more vulnerable to the effect of social isolation as a result of the pandemic. Similar studies should endeavor to allow administration of surveys *via* a variety of means, such as phone or post, to capture the experience of those across the so-called digital divide. Indeed, the included cohort is a biased and nonrepresentative sample of the wider London population. The CCRR cohort are healthier with fewer comorbidities than would be expected for this age-group, are predominantly Caucasian and living within the West London region, an area typically associated with higher socioeconomic status (24). Finally, we found a strong and convincing link between subjective loneliness and higher risk for reporting worsened levels of anxiety and depression. However, this variable warrants a more in-depth investigation, with loneliness being gathered *via* an existing and validated questionnaire designed to assess a wider spectrum of loneliness indicators, such as both emotional and social, believed to be distinct concepts (47). We have, since, optimized our survey questionnaire to capture such additional data.

### Conclusion and Perspectives

The negative impact of COVID-19 on mental health among the general population has been identified as a research priority (1, 2, 6, 33, 48). However, few studies to date have specifically addressed the effect of COVID-19 and consequential social and physical distancing measures on mental wellbeing, specifically among an older population. Findings from this study highlight potentially important clinical and public health implications. We have identified, within an older, UK population, risk factors for the development of anxiety and depression as a result of COVID-19 related social isolation. These factors may inform risk stratification and targeted intervention strategies at both a clinical and community level. We highlight the need to track, identify and implement early interventions among individuals at increased risk of developing loneliness as a result of social isolation. Of the interventions used to combat loneliness and social isolation, effective strategies include those that facilitate engagement in meaningful, satisfying group activities, and psychological interventions to address the maladaptive conditions associated with loneliness (16). As in-person intervention strategies during pandemics may be limited or impossible, the use of technologies, such as apps, may remain an important tool, albeit limited by the digital divide, thus potentially excluding significant numbers of particularly vulnerable older people. These and other adaptive strategies to improve knowledge, awareness and self-coping will be vital in mitigating the risk of loneliness, anxiety and depression in older people.

## Data Availability Statement

The raw data supporting the conclusions of this article will be made available by the authors, without undue reservation.

## Ethics Statement

The CCRR study was ethically approved by the Imperial College London Joint Research Compliance Office (20IC5942) and by the East Midlands Derby Health Research Authority (16/EM/0213). The participants provided their written informed consent to participate in the study.

## Author Contributions

CR, CdJ, SA-A, CU-M, and LM conceptualized and designed the study. CR performed the data analyses with SA-A, CdJ, and CR conducted the literature review. CR wrote the manuscript with co-authors. All authors contributed to the article and approved the submitted version.

## Conflict of Interest

The authors declare that the research was conducted in the absence of any commercial or financial relationships that could be construed as a potential conflict of interest.
